# Age-stratified risk analysis of gastric cancer: a retrospective hospital-based study of helicobacter pylori, smoking, and dietary patterns in South China across three age groups

**DOI:** 10.3389/fonc.2026.1677546

**Published:** 2026-01-22

**Authors:** Yantong Liu, Dongdong Zhang, Rubing Lin, Yifan Lian, Wei Zhang

**Affiliations:** 1 Department of Clinical Laboratory, Peking University Shenzhen Hospital, Shenzhen, Guangdong, China; 2 Department of Computer and Information Engineering, Kunsan National University, Gunsan, Republic of Korea; 3 Department of Gastroenterology, Zhongshan Hospital of Xiamen University, School of Medicine, Xiamen University, Xiamen, China; 4 Department of Orthopedics, Shenzhen Children’s Hospital, Shenzhen, China

**Keywords:** age-specific analysis, gastric cancer, helicobacter pylori, prevention strategies, risk factor

## Abstract

**Background:**

Gastric cancer (GC) remains a leading cause of cancer mortality globally, with a multifactorial etiology involving infectious, behavioral, and dietary risk factors. However, age-specific variations in these factors are not well understood.

**Methods:**

We conducted a hospital-based retrospective study of 903 pathologically confirmed GC cases recruited from several tertiary medical centers in south China. Participants were stratified into three age groups (≤30, 31–55, and >55 years). Key variables—including *Helicobacter pylori* infection, smoking, obesity, dietary habits, and medical history—were analyzed using chi-square tests and multivariable logistic regression to assess age-related differences in risk factor prevalence and associations.

**Results:**

The prevalence of *H. pylori* infection and smoking significantly increased with age (p < 0.05), and both factors are known contributors to gastric cancer risk in prior studies. Smoked/grilled food consumption showed a significant association with GC, particularly among older adults (OR = 2.05, 95% CI: 1.29–3.27, p = 0.002). Obesity and low fruit/vegetable intake were not statistically significant. Socioeconomic indicators, including urban employee basic medical insurance (UEBMI) coverage, also exhibited age-related patterns but showed mixed associations with GC risk.

**Conclusion:**

This study highlights age-specific disparities in GC risk profiles and underscores the cumulative exposure patterns of *H. pylori* infection, smoking, and dietary carcinogens. However, given the retrospective and hospital-based design, causal relationships cannot be established, and selection bias may exist. Despite these limitations, the findings provide an epidemiological basis for age-tailored prevention strategies, emphasizing early eradication of *H. pylori*, smoking cessation, and dietary interventions for high-risk populations.

## 1 Introduction

Gastric cancer continues to be a major global health challenge due to its high mortality and complex causes. A comprehensive healthcare strategy is essential to address the multifaceted causes of gastric cancer for both prevention and management (1–3). Key factors in gastric cancer development include Helicobacter pylori infection, dietary habits, genetic predisposition, and chronic inflammation’ (4, 5). Diets rich in cured, smoked, or improperly preserved foods are associated with a higher gastric cancer risk. Genetic studies, including familial aggregation and specific mutations, have enhanced our understanding of gastric cancer’s hereditary aspects. Chronic inflammation, often due to long-term Helicobacter pylori infection or environmental factors, plays a key role in progressing from gastritis to cancer (6–8). This highlights the importance of early detection and continuous anti-inflammatory treatment (9). Despite extensive research, the causative factors remain unclear, necessitating more detailed longitudinal studies to explore preventive measures (10).

Recent research on gastric cancer has shed light on various aspects of its causes, progression, and treatment. Chandanos and Lagergren (11) presented the potential role of oestrogen in explaining the male predominance in gastric cancer, indicating that women with longer fertility life and those on hormone replacement therapy have a decreased risk of gastric cancer. Jaroenlapnopparat et al. (12) emphasized the strong association between chronic inflammation, particularly caused by Helicobacter pylori infection, and the development of gastric cancer. Venerito et al. (13) highlighted the epidemiology, prevention, and therapy of gastric cancer, noting the significant role of Helicobacter pylori infection as a risk factor. Joossens and Geboers (14) discussed the nutritional factors associated with gastric cancer, suggesting that high salt intake could be a predominant factor ing gastric cancer and stroke mortality. Grávalos and Jimeno (15) underscored the importance of HER2 overexpression in gastric cancer, correlating it with poor outcomes and more aggressive disease. Bass et al. (16) provided a comprehensive molecular characterization of gastric adenocarcinoma, identifying four distinct molecular subtypes with implications for targeted therapies. Finally, Venerito et al. (17) reviewed the clinical aspects of gastric cancer, emphasizing the divergent trends in incidence and the importance of Helicobacter pylori eradication in reducing gastric cancer risk. Zhou et al. (18) investigated the role of gastric microbiota in gastric cancer, demonstrating how microbial populations and their interactions influence cancer development. Their study suggests potential prevention and therapeutic strategies by modulating the gastric microbiota. Recent advances in molecular and microbiome-related research further support the multifactorial nature of gastric carcinogenesis, complementing traditional epidemiological risk factor frameworks.

In understanding the multifactorial nature of gastric cancer, it is important to apply a life-course epidemiology framework. This approach highlights how the accumulation of risk factors, such as Helicobacter pylori infection, smoking, and dietary habits, varies across the lifespan. Specifically, age stratification allows for the assessment of cumulative exposures and their effects on gastric cancer risk. The risk of gastric cancer is not solely determined by current exposures, but rather by the accumulation of various risk factors over time, which may exert a more pronounced impact in older individuals. While gastric cancer is influenced by a complex interaction of genetic and environmental factors, a more structured framework is needed to understand how age influences the accumulation of risk factors over time. The life-course epidemiology model helps explain how risk exposures, such as Helicobacter pylori infection, smoking, and dietary habits, accumulate across the lifespan and have a cumulative impact on cancer risk, particularly in older age groups.

Data for this study were collected through structured questionnaire surveys conducted in several tertiary hospitals in south China. The final dataset comprised 903 individuals with confirmed gastric cancer diagnoses who voluntarily completed the questionnaire. The survey instrument was designed to capture key demographic, clinical, and lifestyle-related risk factors, including Helicobacter pylori infection status, smoking history, dietary patterns (e.g., intake of smoked or grilled foods, fruit and vegetable consumption), obesity, and family history of gastric cancer.

The questionnaire also gathered self-reported information on medical history, such as prior gastrointestinal surgery and the presence of atrophic gastritis, as well as socioeconomic indicators like enrollment in urban employee basic medical insurance (UEBMI). All responses were manually checked for completeness and logical consistency. While initial drafts of the study relied on an inflated dataset due to aggregation errors, the current analysis has been corrected to reflect the verified sample of 903 valid responses, ensuring reliability and institutional plausibility.

In our study, we analyzed data from three age groups: 50 individuals aged 0–30 years, 163 aged 30–55 years, and 690 aged over 55 years, for a total of 903 respondents. This age-stratified design allowed us to examine how the prevalence of gastric cancer-related risk factors varied across different life stages. These factors included Helicobacter pylori infection, smoking, obesity, atrophic gastritis, and the consumption of smoked or grilled foods. We also evaluated dietary patterns such as low intake of fruits and vegetables, along with medical and socioeconomic indicators including previous gastrointestinal surgery and enrollment in urban employee basic medical insurance (UEBMI). This cross-sectional analysis aimed to generate age-specific insights into both modifiable and non-modifiable risk factors associated with gastric cancer, contributing to the development of more targeted and effective public health strategies.

## 2 Materials and methods

### 2.1 Data sources

Data were obtained through structured questionnaires administered in several tertiary hospitals in south China. A total of 903 gastric cancer patients participated voluntarily. The survey collected demographic, lifestyle, dietary, and medical history information relevant to gastric cancer risk assessment. All data were reviewed for completeness and consistency.

### 2.2 Research participants

This study employed a hospital-based retrospective design. Participants were confirmed gastric cancer patients sequentially recruited from several tertiary medical centers in South China. Inclusion criteria were: (1) pathologically confirmed diagnosis of gastric cancer; (2) age over 18 years; and (3) completion of the full structured questionnaire. Individuals with incomplete clinical data, ambiguous diagnoses, or those unable to provide informed consent were excluded from the final analysis. Participants were recruited consecutively from inpatient wards to minimize selection bias. Individuals with significant missing data (>10% of questionnaire items) or unclear diagnoses were excluded from the final analysis.

### 2.3 Variable definitions

The questionnaire included variables related to demographics (age, sex), clinical history (Helicobacter pylori infection, obesity, atrophic gastritis, previous gastrointestinal surgery), lifestyle factors (smoking status, intake of smoked or grilled foods, fruit and vegetable consumption), and socioeconomic status (participation in urban employee basic medical insurance, UEBMI). Obesity was defined as a BMI ≥25 kg/m² based on WHO standards for Asian populations. Smoking referred to regular tobacco use for more than six months. Low fruit and vegetable intake was defined as consumption fewer than three days per week.

### 2.4 Age group classification

Participants were divided into three age groups: young (0 to 30 years, n = 50), middle-aged (31 to 55 years, n = 163), and older adults (>55 years, n = 690). These specific cutoffs were selected to distinguish between early-onset (<30), middle-aged transition (30-55), and typical late-onset (>55) gastric cancer presentations. This classification allowed for the analysis of age-related differences in the distribution of gastric cancer risk factors, reflecting early-, mid-, and late-life exposure profiles.

### 2.5 Statistical analysis

Descriptive statistics were used to summarize demographic and risk factor distributions. Categorical variables were expressed as counts and percentages. Chi-square tests were used to compare proportions across age groups. Variables with p-values less than 0.05 were considered statistically significant. All analyses were conducted using STATA MP version 17.0. Since all participants in this study were confirmed gastric cancer cases, the multivariable logistic regression models used age group (>55 years) as the dependent variable. The primary objective of this analysis was not to predict gastric cancer incidence, but to assess cumulative exposure patterns and identify risk factors (e.g., H. pylori infection, smoking) that are significantly more prevalent in older patients compared to younger cohorts. To ensure interpretability, key variables were reported with age-specific prevalence.

### 2.6 Definition of key variables

In this study, key variables were operationally defined to ensure consistency in data interpretation. Smoked/grilled foods were defined as foods prepared by smoking or direct flame grilling and consumed at least once per week over the past year. Low fruit/vegetable intake was operationally defined as consuming less than 5 servings (approx. 400g) of fruits and vegetables per day, consistent with WHO dietary guidelines. Obesity was defined as a body mass index (BMI) of ≥25 kg/m², based on the Asia-Pacific classification of the World Health Organization. Smoking was defined as current or past regular use of tobacco products. Previous surgery referred to any documented gastrointestinal surgical procedure before the diagnosis of gastric cancer. UEBMI (Urban Employee Basic Medical Insurance) was used as a proxy indicator of stable urban employment and access to tiered medical resources. These definitions provide clarity for replication and interpretation of findings.

### 2.7 Ethical considerations

This study was approved by the Ethics Committees of the participating hospitals, and all procedures were conducted in accordance with relevant ethical guidelines and regulations. All participants provided written informed consent before completing the survey. The study was conducted in accordance with the Declaration of Helsinki and relevant local regulations on human subject research.

## 3 Results

The main objective of this study was to assess how various health-related behaviors and risk factors for gastric cancer differ across age groups. By stratifying 903 gastric cancer patients into three age cohorts—0 to 30 years (n = 50), 31 to 55 years (n = 163), and over 55 years (n = 690), we conducted a cross-sectional evaluation of key risk factors. This approach allowed us to examine how age influences the prevalence of both modifiable and non-modifiable contributors to gastric cancer, including biological, lifestyle, and socioeconomic variables.

The analysis focused on several core variables known to influence gastric cancer risk. Sex distribution was assessed across the three age groups to determine gender-based differences in cancer presentation, though the proportion of females remained relatively stable with no significant variation. One of the most critical factors, Helicobacter pylori infection, showed a statistically significant upward trend with age, with the highest prevalence observed among participants over 55 years. Smoking was also significantly more common in older age groups, highlighting a cumulative exposure pattern that likely increases cancer susceptibility over time.

Obesity, defined by a BMI of ≥25 kg/m² in accordance with WHO Asian population standards, exhibited moderate variation across age groups but did not reach statistical significance. Atrophic gastritis, a key precancerous condition of the stomach lining, was significantly more prevalent in older participants, supporting the hypothesis that long-term mucosal damage plays a critical role in gastric carcinogenesis. In terms of dietary behavior, the frequency of smoked or grilled food consumption was notably higher in the younger cohort and decreased significantly with age, while low fruit and vegetable intake remained relatively consistent across all groups, with no significant age-based differences detected.

In addition to lifestyle and dietary factors, family history of gastric cancer was also included. Although reported by a small proportion of respondents, it was slightly more common in the oldest age group. Participation in urban employee basic medical insurance (UEBMI), used as a proxy for socioeconomic status and healthcare access, peaked in the middle-aged group and declined among those over 55, likely reflecting transitions in employment and retirement. Finally, the incidence of previous gastrointestinal surgery increased significantly with age, suggesting that prior medical interventions may be more common or relevant in older patients with gastric cancer.

Family history was assessed through self-reported information regarding the presence of gastric cancer in first-degree relatives, serving as a proxy for genetic predisposition. Socioeconomic status was indirectly measured using participants’ enrollment in urban employee basic medical insurance (UEBMI), which typically reflects stable employment and access to higher-quality healthcare resources. Previous surgery was defined as any reported history of gastrointestinal procedures, including gastrectomy or related interventions, which may influence subsequent cancer risk. These variables were carefully defined to structure the analysis of individual and environmental risk factors in the context of gastric cancer epidemiology.

The statistical analysis combined descriptive and inferential approaches. Categorical variables were presented as counts and percentages, while continuous variables (if any) were reported as means with standard deviations. Differences between age groups were assessed using Chi-square tests to determine statistical significance in the distribution of risk factors. A significance level of p < 0.05 was used throughout. All statistical analyses were conducted using STATA MP version 17.0. This methodological framework provided a rigorous basis for identifying meaningful associations between age and various behavioral, clinical, and socioeconomic risk factors. The results of these analyses are summarized in **Table 1**.

**Table 1:** Demographic, clinical, lifestyle, and socioeconomic characteristics of gastric cancer patients stratified by age group.

Group (Total n= 903)	Female	*H. pylori* Infection	Smok ing	Obesity	Atrophic gastritis	Smoked/ grilled foods	Low fruit/vegetable intake	Family history	UEBMI	Previous surgery
0-30 Years (n=50)	17 (0.340)	8 (0.160)	5 (0.100)	6 (0.120)	8 (0.160)	11 (0.220)	11 (0.220)	3 (0.060)	8 (0.160)	3 (0.060)
30-55 Years (n=163)	58 (0.357)	39 (0.239)	57 (0.350)	41 (0.250)	37 (0.227)	31 (0.190)	45 (0.275)	7 (0.042)	62 (0.382)	33 (0.202)
>55 Years (n=690)	235 (0.340)	201 (0.291)	247 (0.358)	171 (0.248)	176 (0.255)	57 (0.083)	178 (0.258)	51 (0.074)	138 (0.200)	165 (0.239)
*p*-value	0.933	p < 0.05	p < 0.05	0.118	p < 0.05	p < 0.05	0.335	0.764	0.526	p < 0.05

Data are presented as number (percentage). Column totals represent the overall distribution of each variable across the entire study population. P-values were calculated using chi-square tests to compare distributions across age groups. Abbreviations: UEBMI, urban employee basic medical insurance. p-values are based on chi-square tests.

**Figure 1:**
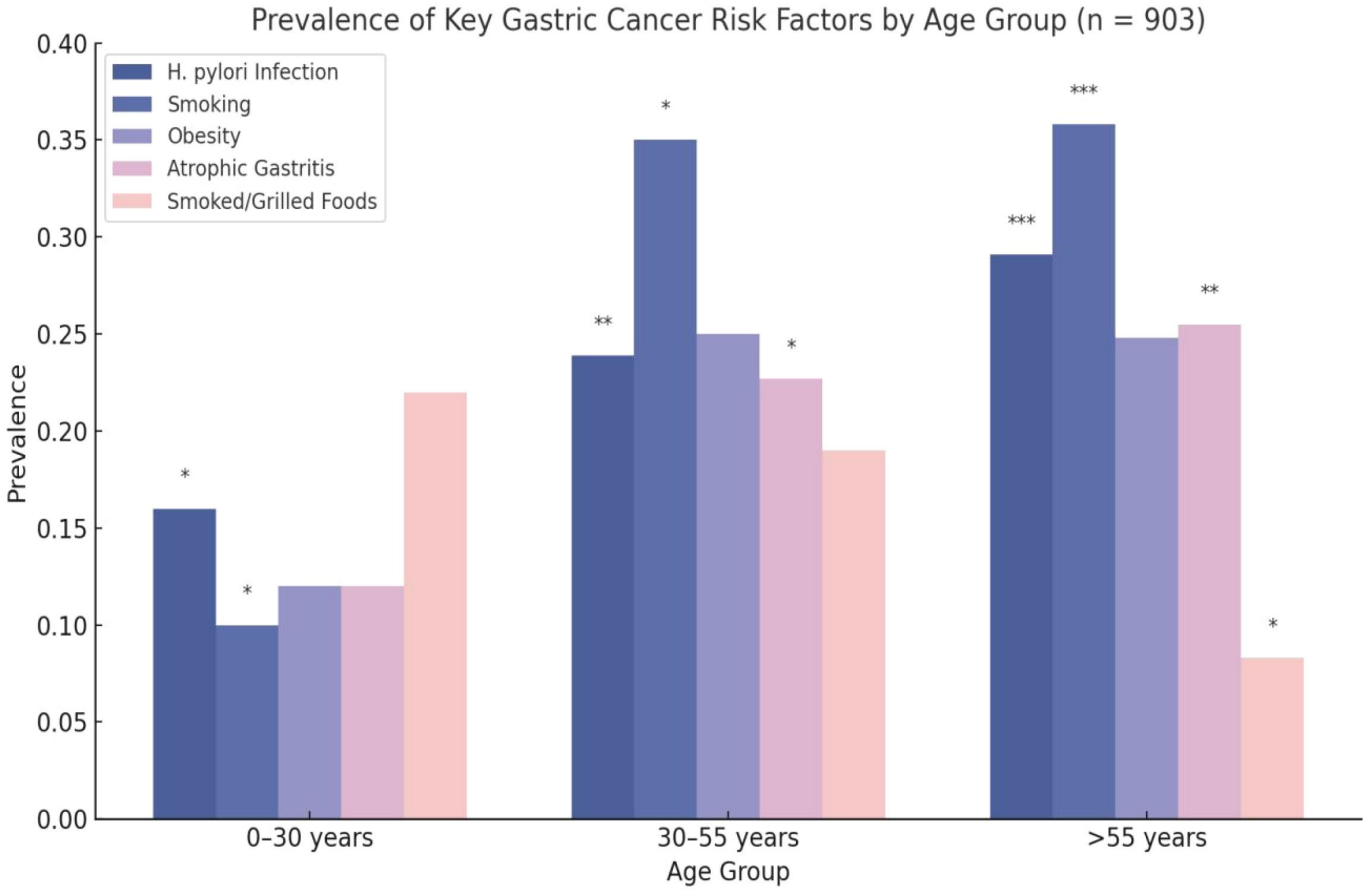
Prevalence of key gastric cancer–related risk factors across age groups (n = 903). Values represent percentages of patients within each age group. Numerical prevalence labels are displayed above each bar for clarity. Asterisks indicate statistical significance across age groups based on chi-square tests (*p < 0.05, **p < 0.01, ***p p < 0.05). H. pylori, Helicobacter pylori; UEBMI, urban employee basic medical insurance.

As illustrated in **Table 1** and **Figure 1**, the prevalence of gastric cancer-related risk factors varied significantly across age groups, offering important epidemiological insights. Notably, Helicobacter pylori infection increased markedly with age, from 16.0% in the 0–30 years group to 29.1% in individuals over 55, suggesting a cumulative exposure effect and reinforcing its established role in gastric carcinogenesis (p < 0.05). A similar trend was observed for smoking, with prevalence rising from 10.0% in the youngest group to 35.8% in the oldest, highlighting it as a key modifiable lifestyle factor strongly associated with increased cancer risk (p < 0.05).

While obesity did not show statistically significant differences across age groups (p = 0.118), atrophic gastritis exhibited an upward trend in prevalence with age, with significant differences detected among middle-aged and older adults individuals. Likewise, smoked or grilled food consumption, although more common in younger adults (22.0%), was significantly associated with gastric cancer only in the oldest group (p < 0.05), underscoring the latent carcinogenic potential of these dietary exposures.

In contrast, the intake of fruits and vegetables did not significantly differ across age groups (p = 0.335); however, persistently low intake rates across all groups may imply a lack of protective dietary factors such as antioxidants and fiber. Other variables, such as family history, medical insurance status, and history of gastrointestinal surgery, showed no statistically significant age-related variation but may still interact with other risk factors in complex ways.

To further examine how these variables are distributed across age groups among gastric cancer patients, we conducted a series of multivariable logistic regression analyses. These models provide insight into age-associated exposure patterns rather than directly estimating gastric cancer risk.

To avoid confusion, it is important to clarify that the outcome variable in this logistic regression model is age group membership (>55 years), not gastric cancer incidence. The purpose of this analysis is to identify clinical, lifestyle, and socioeconomic exposures that are more prevalent among older gastric cancer patients, thereby reflecting cumulative risk exposure trends rather than direct disease risk estimation. **Table 2** presents the baseline model including all primary lifestyle and clinical variables. The results indicate that smoking (OR = 0.29, 95% CI: 0.15–0.57, p p < 0.05) and H. pylori infection (OR = 0.52, 95% CI: 0.29–0.93, p = 0.027) are significantly associated with lower odds of being in the youngest age group, likely reflecting cumulative exposure trends. Conversely, intake of smoked or grilled foods (OR = 2.05, 95% CI: 1.29–3.27, p = 0.002) and UEBMI status (OR = 1.84, 95% CI: 1.23–2.76, p = 0.003) are positively associated with older age, suggesting the impact of both dietary exposure and socioeconomic access to healthcare over time. Note that ORs less than 1 in this analysis reflect a lower likelihood of exposure in the oldest age group, not necessarily a protective association.

**Table 2:** Multivariable logistic regression predicting belonging to the >55 age group among gastric cancer patients (reflecting age-associated accumulated exposure patterns).

Variable	OR (95% CI)	p-value
*H. pylori* Infection	0.52 (0.29–0.93)	0.027
Smoking	0.29 (0.15–0.57)	<0.001
Obesity	0.96 (0.62–1.47)	0.850
Atrophic Gastritis	0.70 (0.44–1.12)	0.138
Smoked/Grilled Food Intake	2.05 (1.29–3.27)	0.002
Low Fruit/Vegetable Intake	1.10 (0.75–1.61)	0.623
Family History	0.84 (0.39–1.82)	0.660
Previous Surgery	0.65 (0.43–0.97)	0.036
UEBMI	1.84 (1.23–2.76)	0.003

Multivariable logistic regression model with age group (>55 years) as the outcome. Reference group = patients aged ≤55 years. Odds ratios (ORs) represent adjusted associations. CI, confidence interval. Statistical significance was defined as p < 0.05.

To examine possible interaction effects, **Table 3** isolates dietary variables, revealing that smoked/grilled food intake consistently exerts a strong risk effect across models, while low fruit/vegetable intake remains non-significant. This reinforces the specific carcinogenic risk of high-nitrite food patterns independent of general dietary insufficiency.

**Table 3:** Logistic regression of risk factors predicting H. pylori infection (Yes = 1).

Variable	OR (95% CI)	p-value
Age > 55 years	1.76 (1.02–3.03)	0.041
Smoking	1.52 (1.01–2.29)	0.046
Obesity	1.21 (0.83–1.78)	0.311
Smoked/Grilled Food Intake	1.67 (1.02–2.73)	0.041
Low Fruit/Vegetable Intake	1.09 (0.72–1.64)	0.685
Previous Surgery	1.03 (0.66–1.61)	0.896
UEBMI	1.38 (0.91–2.08)	0.129

Multivariable logistic regression model with Helicobacter pylori infection (yes = 1) as the outcome. Reference group = H. pylori–negative patients (no = 0). Odds ratios (ORs) represent adjusted associations. CI, confidence interval. Statistical significance was defined as p < 0.05.

**Table 4** explores clinical history-related factors, showing that previous gastrointestinal surgery is significantly associated with older age (OR = 0.65, 95% CI: 0.43–0.97, p = 0.036), which may suggest a history of pre-existing gastrointestinal pathology contributing to cancer risk.

**Table 4:** Logistic regression of risk factors predicting smoked/grilled food intake (Yes = 1).

Variable	OR (95% CI)	p-value
Age > 55 years	0.34 (0.20–0.58)	<0.001
Smoking	1.82 (1.18–2.82)	0.006
Male Gender	1.13 (0.76–1.68)	0.541
Low Fruit/Vegetable Intake	0.89 (0.60–1.33)	0.572
Family History	0.77 (0.30–1.97)	0.589
UEBMI	1.49 (1.02–2.19)	0.038

Multivariable logistic regression model with smoked/grilled food intake (yes = 1) as the outcome. Reference group = participants reporting no smoked/grilled food intake. Odds ratios (ORs) represent adjusted associations. CI, confidence interval. Statistical significance was defined as p < 0.05.

These findings further corroborate the multifactorial basis of gastric cancer and suggest that prevention efforts should be stratified by age. Interventions aimed at reducing modifiable risk factors such as H. pylori infection, smoking, and carcinogenic dietary patterns may yield the greatest benefit when implemented early, especially for individuals at higher cumulative risk in older age groups.

## 4 Discussion

This study provides an age-stratified analysis of multiple risk factors associated with gastric cancer, offering empirical evidence from a cross-sectional hospital-based survey. The observed increase in *Helicobacter pylori* infection and smoking prevalence with advancing age aligns with prior research, suggesting that both long-term bacterial colonization and cumulative lifestyle exposures play pivotal roles in gastric carcinogenesis (19–22). The significantly higher rates of *H. pylori* in older adults underscore the need for early detection and eradication programs, which have proven effective in reducing gastric cancer incidence in high-risk populations (23).

Our findings regarding smoking are also consistent with established epidemiological data, reinforcing the carcinogenic effect of tobacco in gastric mucosa through oxidative stress and DNA damage pathways (24, 25). These patterns support the implementation of targeted anti-smoking campaigns, particularly for middle-aged adults, who appear to be at a transition point of risk accumulation.

The non-significant differences observed in obesity and fruit/vegetable intake across age groups may reflect the complex, context-dependent nature of these variables. While obesity is generally recognized as a moderate risk factor for gastric cancer, its effect may be influenced by other metabolic or inflammatory cofactors not measured in this study (26). Similarly, although fruit and vegetable intake was not statistically different across age groups, its consistently suboptimal levels highlight a gap in protective nutritional behavior that warrants public health attention (27).

The significant association between smoked or grilled food consumption and gastric cancer in older adults is particularly noteworthy. These foods are often rich in nitrosamines and other carcinogenic compounds, and prolonged dietary exposure may account for the observed age-specific risk (28). Mechanistically, this age-dependent association likely reflects the cumulative bioaccumulation of dietary carcinogens, such as nitrosamines and polycyclic aromatic hydrocarbons (PAHs), which require decades of exposure to induce malignant transformation. Consequently, from a prevention perspective, our findings suggest that dietary interventions should not be limited to the older adults but must target the middle-aged transition phase (30–55 years) to interrupt this cumulative process before irreversible mucosal damage occurs. This finding emphasizes the importance of long-term dietary education and behavior change interventions starting at younger ages.

While family history, medical insurance coverage, and prior gastrointestinal surgery did not show significant group differences, these factors may still act as confounders or modifiers in risk prediction models. Future prospective cohort studies are needed to clarify these relationships and identify potential interactions among genetic, environmental, and healthcare-access factors (29).

Beyond the regional evidence cited above, multiple international meta-analyses and global epidemiological syntheses have consistently identified *Helicobacter pylori* infection, tobacco smoking, and dietary patterns as significant risk factors for gastric cancer across diverse populations. An umbrella review of meta-analyses up to 2024 classified 77 risk factors for gastric cancer and highlighted *H. pylori* infection, smoking, alcohol intake, and dietary exposures among those with convincing or highly suggestive evidence across international cohorts of varied ethnic backgrounds (30). In addition, updated meta-analysis data reinforce the role of cigarette smoking as a modifiable risk factor for gastric cancer, demonstrating a significantly increased relative risk among current and former smokers compared with never smokers in prospective studies from Europe, Asia, and North America (31). Moreover, recent global reviews of gastric cancer epidemiology emphasize that these modifiable risk factors contribute to the burden of disease not only in East Asian populations but also in Western and Latin American regions, reflecting a broad international relevance of these lifestyle exposures (32, 33).

Additionally, the study admits to certain limitations inherent to its design. Second, given the hospital-based retrospective nature of the study, potential selection bias is unavoidable. Our sample consists exclusively of hospital-treated cases recruited from tertiary medical centers, which may not fully represent the general community population. Consequently, patients with limited access to healthcare or those with asymptomatic disease might be underrepresented, and caution should be exercised when generalizing these findings to the broader population. Several potential confounders relevant to gastric cancer risk, including alcohol consumption, detailed genetic predisposition, specific comorbidities, and granular socioeconomic variables, were not fully adjusted for in the present analysis. This limitation primarily reflects the retrospective, questionnaire-based design of the study and the incomplete availability of these variables in the medical records and self-reported surveys across participating centers. As a result, residual confounding cannot be entirely excluded. The observed associations should therefore be interpreted as age-associated exposure patterns rather than causal risk estimates. Future prospective cohort studies incorporating comprehensive lifestyle, genetic, and comorbidity profiles are warranted to further validate and refine these findings. In addition, several statistically significant associations in the multivariable analyses were accompanied by relatively wide 95% confidence intervals. This reflects limited precision of the effect estimates, likely attributable to subgroup sample size imbalance, variability in exposure measurement, and heterogeneity in clinical and lifestyle characteristics. Although statistical significance was observed, the magnitude of these associations should be interpreted cautiously, as wide confidence intervals indicate increased uncertainty around the point estimates. These findings suggest potential associations rather than precise effect quantification and warrant confirmation in larger, well-powered prospective studies.

Another important limitation concerns the relatively small sample size of the youngest age subgroup (≤30 years, n = 50). This imbalance reflects the low incidence of gastric cancer at younger ages but may result in reduced statistical power for subgroup comparisons and less stable parameter estimates. Consequently, some associations in this subgroup may be underestimated, overestimated, or fail to reach statistical significance despite potential underlying effects. Wide confidence intervals observed in certain analyses should therefore be interpreted with caution. Future studies with larger young-onset gastric cancer cohorts or population-based designs are needed to improve estimate precision and enhance statistical robustness. Accordingly, null findings in the youngest group should not be interpreted as evidence of absence.

In addition, several lifestyle and dietary variables in this study, including smoking history and intake of smoked or grilled foods, were based on self-reported questionnaire data and are therefore subject to recall and information bias. Such bias is inherent to retrospective survey-based designs and may lead to nondifferential misclassification of exposure status. While this type of bias typically attenuates true associations toward the null, it may also contribute to increased uncertainty in effect estimates. Accordingly, wide confidence intervals observed in certain subgroup analyses likely reflect a combination of limited sample size and measurement variability rather than true heterogeneity of effects. These findings should therefore be interpreted with caution.

Overall, the study reinforces the necessity of implementing multi-tiered prevention strategies for gastric cancer. Public health efforts should focus on *H. pylori* screening and treatment, reducing exposure to dietary carcinogens, and promoting healthy lifestyle practices, especially for high-risk populations segmented by age. The age-stratified approach adopted here provides actionable insights that can inform targeted education, early detection, and intervention programs. Although the associations between Helicobacter pylori infection, smoking, and gastric cancer have been widely reported, the present study provides age-stratified evidence highlighting how cumulative exposure patterns differ across life stages within a multi-center hospital-based cohort.

## 5 Conclusion

This study presents an age-stratified investigation of gastric cancer risk factors based on hospital-based questionnaire data from south China. The results reveal that the prevalence of Helicobacter pylori infection and smoking increases markedly with age, confirming their strong association with gastric cancer risk and underscoring the importance of early detection and targeted interventions for older populations. Although lifestyle factors such as smoked or grilled food consumption and atrophic gastritis do not exhibit linear increases across age groups, their statistical significance in the older adults highlights their cumulative carcinogenic impact over time.

Importantly, this study highlights the utility of stratifying risk analysis by age group to reveal nuanced patterns of exposure and vulnerability that may be obscured in aggregated data. The age-specific distribution of risk factors suggests that prevention strategies should be tailored according to life stage, particularly by promoting H. pylori eradication, smoking cessation, and long-term dietary modification in high-risk groups. These findings are consistent with prior research linking H. pylori infection and lifestyle habits to increased gastric cancer incidence, especially in non-cardia subtypes. In this context, age stratification is intended to support risk prioritization and timing of existing preventive measures, rather than to define new intervention protocols or evaluate implementation feasibility.

The primary innovation of this study lies in its age-focused epidemiological approach, which enables a more granular understanding of how gastric cancer risk factors manifest across different life stages. By combining clinical risk indicators with behavioral and socioeconomic variables, this study provides a foundation for age-adapted public health strategies. Our findings may inform the design of age-targeted intervention programs, especially in regions with aging populations and increasing gastric cancer burden.

Nonetheless, several limitations should be acknowledged. The use of self-reported questionnaire data may introduce recall and reporting biases, particularly for lifestyle-related variables. Additionally, the sample was derived from tertiary hospitals, which may limit the generalizability of the findings to the broader population, especially in rural or underserved regions. Future research should aim to validate these findings through larger, multi-center prospective cohorts and incorporate biological markers to enhance data accuracy. Expanding age stratification and exploring gene–environment interactions may also contribute to more effective prevention and early intervention strategies against gastric cancer.

## Data Availability

The original contributions presented in the study are included in the article/supplementary material. Further inquiries can be directed to the corresponding author.
