# Correction: Age-stratified risk analysis of gastric cancer: a retrospective hospital-based study of helicobacter pylori, smoking, and dietary patterns in South China across three age groups

**DOI:** 10.3389/fonc.2026.1808943

**Published:** 2026-02-23

**Authors:** Yantong Liu, Dongdong Zhang, Rubing Lin, Yifan Lian, Wei Zhang

**Affiliations:** 1 Department of Clinical Laboratory, Peking University Shenzhen Hospital, Shenzhen, Guangdong, China; 2 Department of Computer and Information Engineering, Kunsan National University, Gunsan, Republic of Korea; 3 Department of Gastroenterology, Zhongshan Hospital of Xiamen University, School of Medicine, Xiamen University, Xiamen, China; 4 Department of Orthopedics, Shenzhen Children’s Hospital, Shenzhen, China

**Keywords:** age-specific analysis, gastric cancer, helicobacter pylori, prevention strategies, risk factor

The funders Shenzhen High-level Hospital Construction Fund, and Peking University Shenzhen Hospital Scientific Research Fund (KYQD2025465) to Wei Zhang were erroneously omitted.

The correct **Funding** statement reads:

“The author(s) declared that financial support was received for this work and/or its publication.This research was funded by the National Natural Science Foundation of China (Grant No: 82303109), Natural Science Foundation of Fujian Province (Grant No: 2022J05299), Kunsan National University’s Industry-Academia Cooperation Group (NO. 2023H052), Shenzhen High-level Hospital Construction Fund, and Peking University Shenzhen Hospital Scientific Research Fund (KYQD2025465)”.

The original version of this article has been updated.

